# Supplementary figures and images for: IPSC-Derived Astrocytes Contribute to In Vitro Modeling of Parkinson’s Disease Caused by the GBA1 N370S Mutation
Source: Int J Mol Sci. 2023 Dec 26;25(1):327. doi: 10.3390/ijms25010327 (PMC10779194; doi:10.3390/ijms25010327)

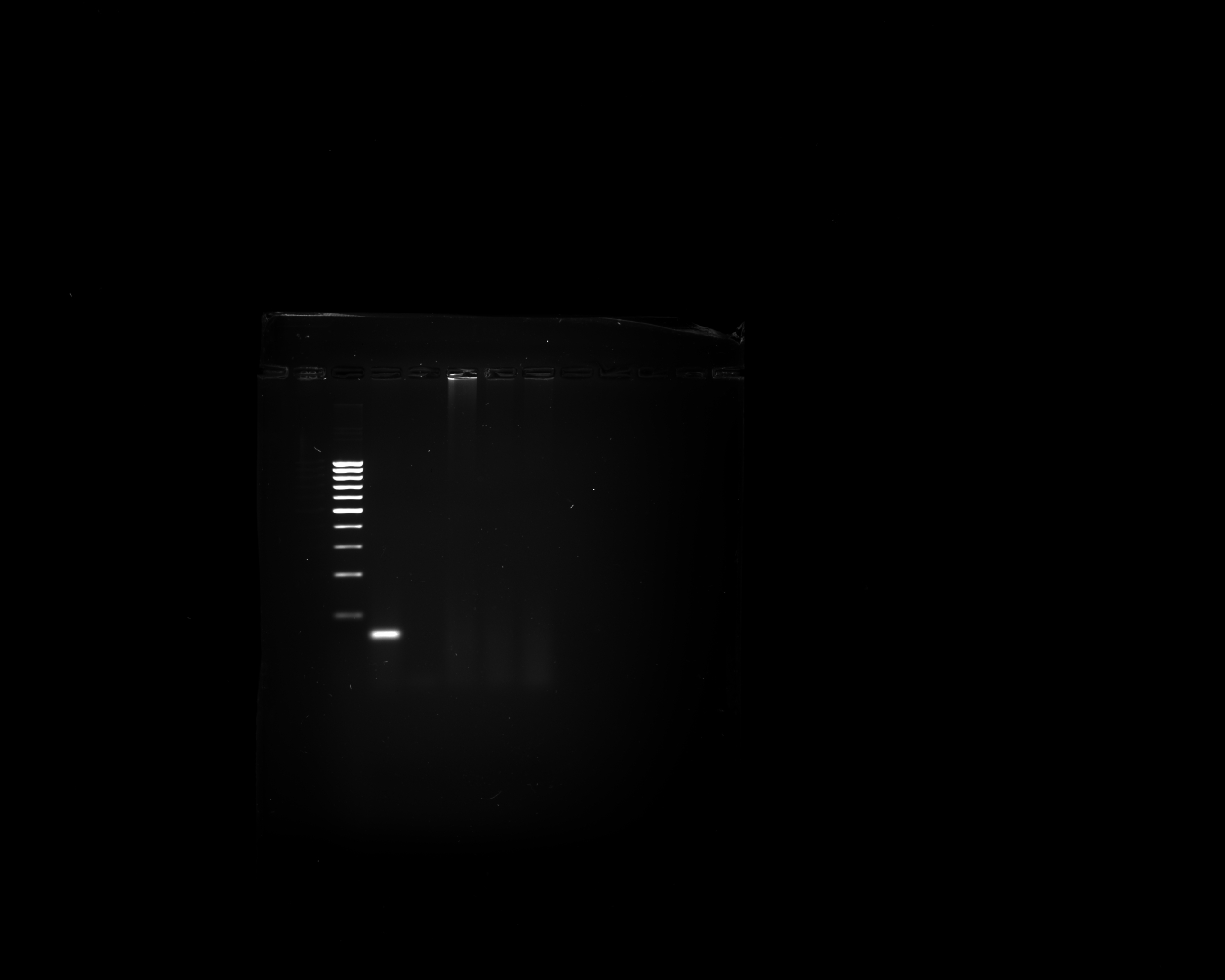

Supplement: Supplementary file 1 [file ijms-25-00327-s001.zip › epi.jpg]

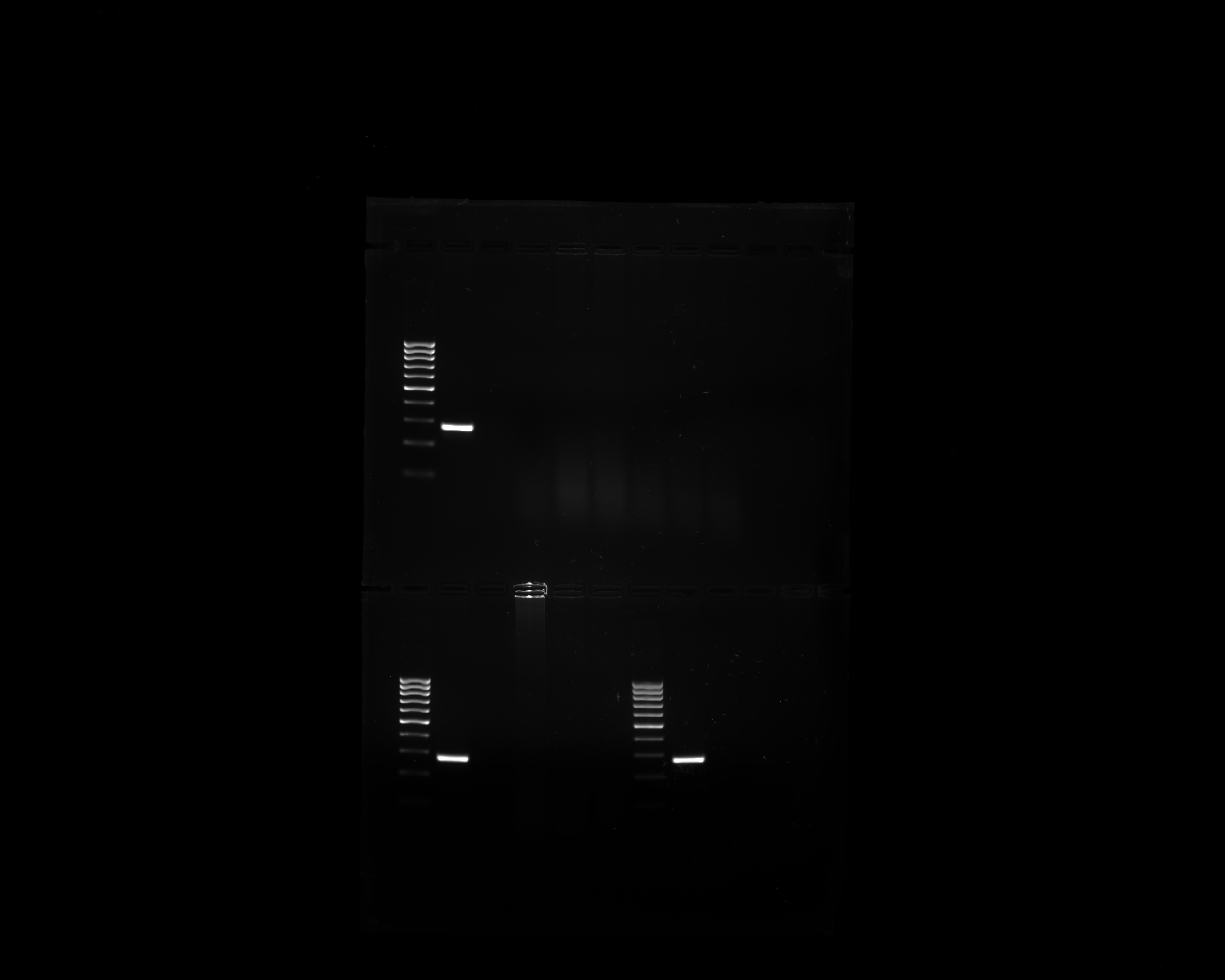

Supplement: Supplementary file 1 [file ijms-25-00327-s001.zip › myco.jpg]

## Supplementary Materials

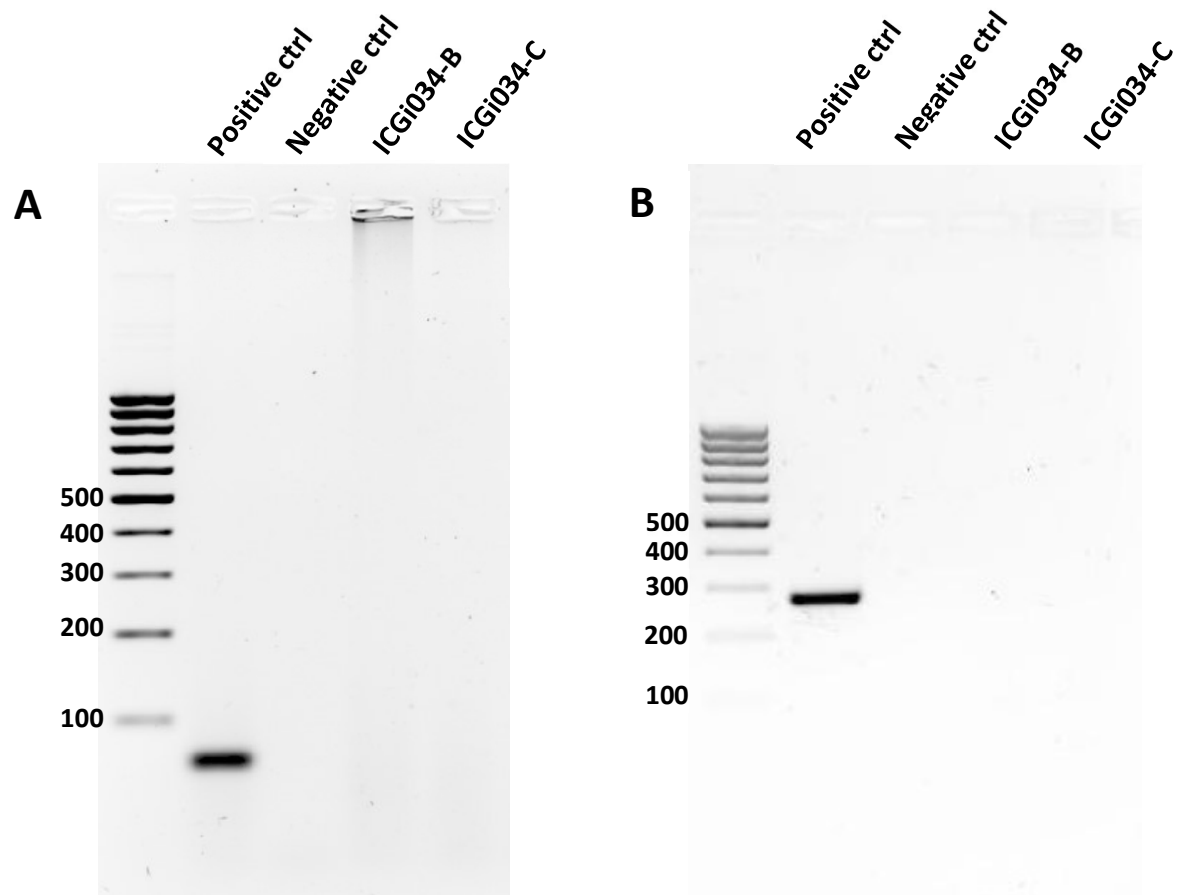

**Figure S1.** Episomes (A) and mycoplasma (B) detection.

Supplement: Supplementary file 1 [file ijms-25-00327-s001.zip › Supplementary Material FS1.pdf]
